# Cardiac Conduction Velocity, Remodeling and Arrhythmogenesis

**DOI:** 10.3390/cells10112923

**Published:** 2021-10-28

**Authors:** Bo Han, Mark L. Trew, Callum M. Zgierski-Johnston

**Affiliations:** 1 Institute for Experimental Cardiovascular Medicine, University Heart Center Freiburg-Bad Krozingen, 79110 Freiburg im Breisgau, Germany; bo.han@uniklinik-freiburg.de; 2 Faculty of Medicine, University of Freiburg, 79110 Freiburg im Breisgau, Germany; 3 Spemann Graduate School of Biology and Medicine (SGBM), University of Freiburg, 79104 Freiburg im Breisgau, Germany; 4 Department of Cardiovascular Surgery, The Fourth People’s Hospital of Jinan, 250031 Jinan, China; 5 Auckland Bioengineering Institute, University of Auckland, Auckland 1010, New Zealand; m.trew@auckland.ac.nz

**Keywords:** conduction velocity, arrhythmogenesis, methodology, anisotropy, inhomogeneity, gap junction, whole heart, cardiac remodeling

## Abstract

Cardiac electrophysiological disorders, in particular arrhythmias, are a key cause of morbidity and mortality throughout the world. There are two basic requirements for arrhythmogenesis: an underlying substrate and a trigger. Altered conduction velocity (CV) provides a key substrate for arrhythmogenesis, with slowed CV increasing the probability of re-entrant arrhythmias by reducing the length scale over which re-entry can occur. In this review, we examine methods to measure cardiac CV in vivo and ex vivo, discuss underlying determinants of CV, and address how pathological variations alter CV, potentially increasing arrhythmogenic risk. Finally, we will highlight future directions both for methodologies to measure CV and for possible treatments to restore normal CV.

## 1. Introduction

Cardiac arrhythmias can be defined as any disruption to normal cardiac rhythm. They range from relatively simple changes in normal heart rate to complex, multifactorial fibrillation events. The clinical presentation of arrhythmias varies widely, from asymptomatic or mild disease to severe physical limitations and sudden cardiac death. Major arrhythmic events (such as atrial fibrillation or ventricular tachycardia) have profound effects on quality of life and mortality. Atrial fibrillation, for example, affects more than 30 million people worldwide and will be increasingly prevalent with aging populations [1]. Atrial fibrillation often leads to adverse consequences, such as stroke and heart failure (HF), increasing mortality [2].

In most pathological conditions, arrhythmias are accompanied by structural and electrical remodeling, which tend to be intertwined. Mechanical changes can also result in altered electrical activity at cellular and tissue levels. These alterations may then result in electrical remodeling, which is typically described as a progressive change in the electrophysiological properties of the myocardium. For example, several studies have shown that the electrophysiology (EP) of cells and tissue isolated from failing hearts is characterized by prolonged action potential duration (APD) and reduced conduction velocity (CV). On the other hand, changes in intercellular electrical coupling and tissue makeup contribute to the heterogeneity of APD and CV reduction [3]. 

CV and APD (via effects on refractoriness) are significant contributors to arrhythmogenesis. CV alterations play a major role in the generation and maintenance of cardiac arrhythmias. A voltage optical mapping study in isolated hearts showed that the induction of ventricular fibrillation (VF) at high activation frequencies is associated with CV reduction [4]. Experimental and computational studies further illustrate that the initiation of arrhythmias may be a result of heterogeneity in local CV [5,6,7]. 

CV is of particular interest in understanding determinants of re-entrant arrhythmias. Re-entry typically underlies tachyarrhythmias and fibrillation. Re-entrant activity occurs when excitation fails to stop following the normal cardiac sequence and instead re-excites regions of the heart in advance of the next sinus beat. For re-entry to occur, the tissue must no longer be refractory by the time the excitation wave returns to a given point. This concept is formally defined as the wavelength of the heart, which is equal to the CV multiplied by the refractory period. It was first discussed in 1913 [8], with the idea that in a healthy heart, the ‘wave of excitation’ was sufficiently broad (long wavelength) and fast (large CV) that the ventricle activated once each sinus beat. As the wavelength shortens (through slowed CV and/or accelerated repolarization), it becomes possible that multiple excitation waves can co-exist, re-exciting tissue multiple times without initiation of a new sinus excitation. In short, a reduced wavelength means that re-entry can occur over a smaller distance, thus increasing the likelihood of self-sustained arrhythmias. 

Assessment of cardiac wavelength and of the risk of re-entry requires the accurate measurement of CV. CV is altered by a multitude of factors, all of which may contribute to arrhythmogenesis. To investigate the mechanisms of CV-related arrhythmias, we need to understand how CV is assessed, what the determinants of CV are, and how remodeling and CV interact with one-another. In this review, we focus on current methods of CV measurement in the whole heart in vivo and ex vivo and on physiological and pathological factors that can affect CV and, thus, arrhythmia susceptibility. Compounds utilized for studying the effects of CV and clinical drugs that may affect CV are also discussed.

## 2. Methods of CV Measurement

**Figure 1:**
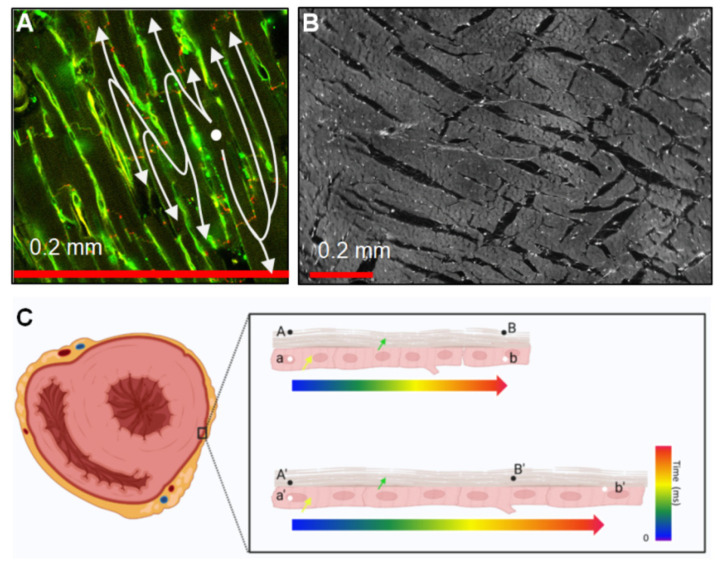
Structure and mechanical considerations affecting CV measurements. (**A**) Cell-to-cell connectivity through intercalated disks located at cell endpoints causes a ‘zig-zag’ conduction in the trans-cell direction that increases path lengths and slows the apparent (extrinsic) conduction [9]. The image shows rat ventricular tissue labeled with wheat-germ agglutin (green) and Cx43 (red). (**B**) Layers are approximately 5–6 cells thick and form laminae orthogonal to the cell long axes. These can also cause increased activation path lengths due to indirect conduction at this scale [10]. The image shows midwall tissue from a rat’s left ventricle. (**C**) Extrinsic and intrinsic CV. The left panel shows a pattern of ventricular transverse sections. The yellow arrow indicates localized cardiomyocytes, and the green arrow shows epicardium. A/A’ and B/B’ indicate spatially fixed points between which the extrinsic CV is measured; a/a’ and b/b’ indicate defined points of the tissue between which the intrinsic CV would be assessed. The top right panel indicates that in the un-stretched myocardium, B and b are depolarized at the same time (intrinsic = extrinsic CV); in the bottom right panel, assuming that the intrinsic CV remains constant, extrinsic CV would appear to be increased as tissue at B’ completes depolarization at an earlier time.

For the study of arrhythmias and the mechanisms of electrical remodeling, precise measurement of CV is an important factor. The basis for measuring CV is relatively simple: either one measures the time required for an electrical impulse to travel a certain distance or the distance traveled in a predefined period of time. However, the curved surface of the heart, transmural (volume conduction) effects, and issues accessing the heart render measurement of cardiac CV non-trivial. Furthermore, since the heart is orthotropic, ideally, multiple CVs are measured, reflecting longitudinal and transverse (relative to locally prevailing cell orientation) CV (CV_l_ and CV_t_), meaning more than two measurement points are necessary. Additionally, CV is, at the scales relevant to cardiac measurements, an emergent property reflecting the interaction of multiple activation paths at smaller scales. If the notional CV of a single cell membrane could be measured, this would be much faster than that of a series of cells (due to ‘delays’ in conduction at cell–cell connections). Expanding this to the tissue scale, inhomogeneity of cell–cell coupling, tissue morphology, and presence of fibrosis means that while we typically think of excitation as a homogenous wave, activation follows tortuous paths through the cell networks (Figure 1A) and the tissue (Figure 1B). CV measurements will, therefore, depend on the scale at which it is measured.

An additional complication is the definition of CV with respect to the underlying tissue. ‘Intrinsic’ or ‘extrinsic’ CV can be distinguished, where intrinsic CV is the speed of conduction between defined anatomical points, and extrinsic CV is the speed of conduction over a set distance. To illustrate this, if it takes a certain amount of time for excitation to travel a length of the myocardium and, presuming no effect of stretch on conduction, if the tissue is stretched, the intrinsic (biological) CV will remain constant, while the extrinsic (biophysical) CV will increase (Figure 1C). Extrinsic CV is most commonly observed; however, this is rarely explicitly stated, and care should be taken when interpreting results.

There are several methods for measuring CV in a whole heart. Optical mapping (OM) of transmembrane voltage is widely used for isolated mammalian hearts; however, application to human and in vivo heart models is limited. Multi-electrode arrays (MEA) are applied both ex vivo and in vivo; however, they are highly invasive, limiting their utility. In clinical settings, electrode mapping catheters are a key method to obtain electrophysiological data, including CV. Finally, electrocardiographic imaging (ECGI) has relatively recently been implemented clinically for the non-invasive assessment of cardiac electrophysiology. How these methods can be used to measure CV, and the pros and cons thereof will be discussed in the following sections.

### 2.1. Optical Mapping

In the laboratory setting, OM is a technique widely adopted for CV measurements as it enables the recording of electrical activity in isolated mammalian hearts at a high spatio-temporal resolution [11,12,13]. OM requires hearts with a voltage-sensitive reporter (either a voltage-sensitive dye or a genetically expressed voltage-sensitive protein), which undergo changes in their fluorescence in response to changes in transmembrane voltage. With the use of appropriate light sources, filters, and cameras, the electrical activity can be optically recorded, usually from the sub-epicardium, allowing observation of activation and repolarization timing as well as action potential (AP) shape. This is used to quantify parameters, such as CV, APD, and arrhythmia dynamics. 

To study arrhythmias in a laboratory environment, typically, a single projection (e.g., anterior or posterior surface of the heart) is imaged [14]. This has been valuable in measuring regional cardiac APD and CV in normal sinus rhythms and during the development and presence of arrhythmias, identifying crucial processes, such as wave block, focal activity, re-entrant rotors, and their phase singularities [14,15]. The acquisition of transmural dynamics and electrical activity from inside cardiac muscle can also be partially done by using simultaneous OM from epi and endocardial sides or putting the light source and camera on the opposite sides of the preparation, which enables the detection of light escaping from layers deep within the tissue; however, these approaches are typically destructive to ensure access to both sides [16,17].

The heterogeneity of CV across the heart, particularly in diseased tissue, means that panoramic OM systems that image the heart from multiple sides are preferable [18,19]. By imaging multiple, often partially overlapping, projections of the heart, the transmembrane voltage can be assessed across most of the epicardial surface (though usually missing atrial and apical projections). On this basis, heterogeneities in EP can be assessed, and sites of focal activation, excitation breakthrough, and conduction block, as well as re-entrant rotors and fibrillatory waves, can be continuously tracked and monitored as they meander across the sub-epicardial myocardium [20,21,22]. 

**Figure 2:**
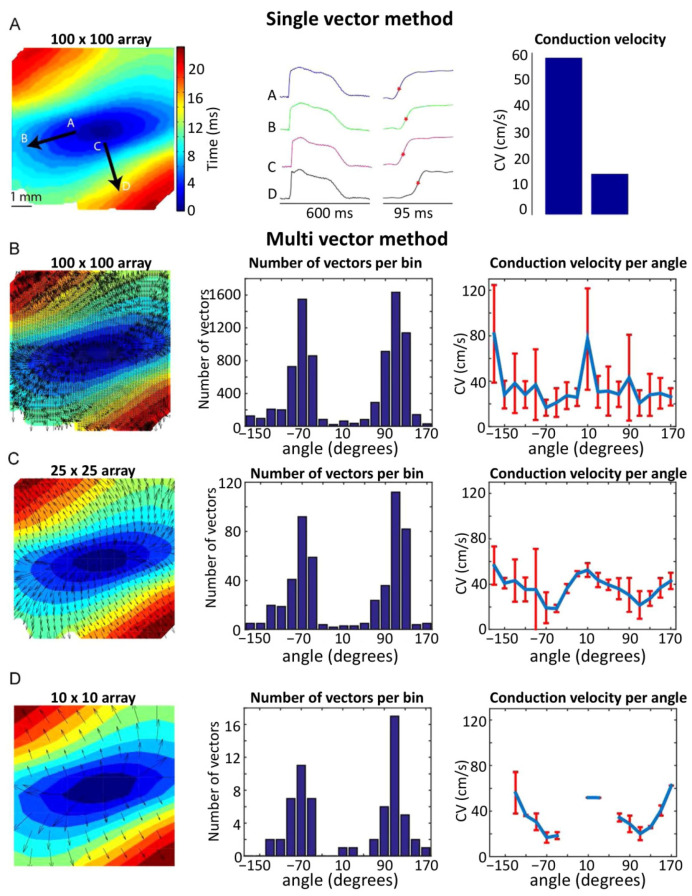
Single- and multi-vector methods for calculating CV. (**A**) (Left panel) Activation map optically recorded from the sub-epicardium of a left ventricular wedge preparation from a human heart during central point-stimulation. Black arrows indicate the directions in which either longitudinal (A–B arrow) or transverse (C–D arrow) CV was measured. (Middle panel) Optically recorded AP from the regions indicated on the activation map, with activation times (time of fastest action potential upstroke) noted. (Right panel) Longitudinal and transversal CV, measured using the single-vector method. (**B**) (Left panel) Activation map of A with superimposed vectors, as calculated by the multi-vector method using 100 × 100 individual analysis regions (Middle panel). Histogram indicating the number of vectors per direction. (Right panel) Average CV for each direction, calculated using the multi-vector method. (**C**,**D**) show data as in B, but for a 25 × 25 array and a 10 × 10 array, respectively. Reproduced with permission from Doshi, A.N. et al. Feasibility of a semi-automated method for cardiac conduction velocity analysis of high-resolution activation maps. *Comput. Biol. Med.* **2015**, *65*, 177–183, doi:10.1016/j.compbiomed.2015.05.008 (accessed on 26 January 2021).

The calculation of CV from OM data, whether a single view or panoramic, can be done using the so-called ‘single vector’ method (although it usually utilizes two vectors; Figure 2). The heart is typically electrically stimulated at a point on the epicardium. Due to differences in longitudinal (along the locally prevailing cell orientation) and transverse CV (perpendicular to cell axes), an activation time map can be generated to ascertain the direction of fastest conduction, assumed to represent the longitudinal direction. The single vector method then calculates CV using the difference in activation times and the distance between two selected points along and perpendicular to the apparent longitudinal axis (Figure 2A) [23]. This method assumes that the direction of transverse propagation lies perpendicular to the globally determined longitudinal direction, which may be incorrect due to complex tissue geometry and the presence of discontinuities. In addition, the orientation of the fastest propagation can be influenced by both the subjective placement of the electrical stimulation point and the selection of points for the single vector method [24]. An alternative is the multi-vector method, where a local velocity vector is computed at each recording point and then binned based on its direction. (Figure 2B–D) [25]. The bin with the largest number of vectors indicates the transverse direction, while the bin with the largest average vector amplitude indicates the longitudinal direction. High (Figure 2B) and low (Figure 2D) spatial resolutions may result in an overestimation of the longitudinal CV by the multi-vector method. An additional potential drawback of this method is the fact that vectors are calculated over a shorter distance, making them more susceptible to noise while simultaneously lowering the temporal resolution. The single vector method uses longer vectors, and therefore, reduces the effects of noise and may increase accuracy; however, it may also obfuscate the actual CV by ignoring the presence of indirect conduction paths between two points. 

One key issue with both approaches is that they assume the physical distance between pixels to be identical across the image, which is only the case when imaging a flat surface. When imaging the whole heart, its surface curvature should be taken into account. Therefore, reconstructions of the three-dimensional (3D) epicardial surface are necessary. This enables the calculation of CV by fitting a local polynomial surface to neighboring activation times, using spherical coordinates rather than the linear coordinates of the projection onto flat detectors, greatly improving the accuracy of CV measurements.

A limitation of most OM-based measurements is the use of excitation-contraction uncouplers. The beating of the heart generates motion-induced imaging artifacts in the OM signals. Movement can be reduced using pharmacological uncouplers that, ideally, do not alter EP. Blebbistatin is the most commonly used uncoupler. It works by preventing cross-bridge formation between actin and myosin filaments and so eliminates active tension generation without major effects on intracellular calcium ([Ca^2+^]_i_) dynamics [26]. While initial studies suggested few, if any, side effects of blebbistatin on EP [11], more recent studies have highlighted changes in APD, CV, and fibrillation dynamics [27,28,29]. The observed changes in EP, however, may not be a direct effect of blebbistatin but rather a consequence of the metabolic state of the heart when adenosine triphosphate is abundant due to reduced utilization for contraction. An additional drawback of excitation-contraction uncouplers is that they prevent the simultaneous study of mechanics, EP, and their mutual interactions. Furthermore, uncouplers are not viable for in vivo OM.

An alternative is the use of motion tracking techniques, which enables the OM of beating hearts. These use motion tracking algorithms to follow either marker placed on the surface of the heart or features apparent from native tissue contrast (possible due to the difference in signal features between the signal generated by changes in membrane voltage and those from motion). Once the motion is known, this can be used to track the fluorescence over time of each point as it moves in space. Additional correction can be performed through the use of ratiometric imaging, where two absorption or emission wavelengths are used so that two signals are obtained, one that increases with increasing membrane potential and one that decreases. By dividing one signal by the other, the effects from changes in illumination parameters and any residual motions can be minimized [30,31], and calculating the fluorescence ratio can eliminate the motion signal. However, these motion tracking algorithms are typically time-consuming and processing-intensive. An encouraging alternative, well-suited for in vivo use, are fiber arrays that can be placed onto the surface of the heart. These move with the heart and, when combined with voltage ratiometry of near-infrared dyes, enable high-resolution cardiac electrophysiology OM, even in vivo [29]. As a well-established laboratory measurement, OM is widely used to measure intrinsic CV, transmembrane potentials, activation, repolarization, and APD shape.

### 2.2. Multi-Electrode Arrays

In the early days, the plunge-needle electrodes containing two to eight bipolar electrode pairs per needle were used to obtain mapping data. With sufficient numbers of electrodes, high-resolution activation maps, including transmural data, could be obtained, but the use of the plunge electrodes is tissue-damaging and hinders motions [32]. Instead, mapping can be performed just from the epi or endocardial side. The traditional method of mapping epicardial activation was to use a handheld movable electrode with a unipolar or bipolar recording tip. The local activation time of each point of interest was measured relative to a fixed reference point. This technique required 5–15 min to acquire the data needed to construct a map. Since each epicardial point was recorded during a separate cardiac cycle, the technique requires a stable rhythm so that activation timings are not significantly different from cycle to cycle. This technique is not suitable, therefore, for studying transient events or unstable activation sequences (for example, during arrhythmias). 

The simultaneous use of multiple electrodes, arranged in a grid or array pattern (multi-electrode array; MEA), enables much faster data acquisition. The first application of an MEA to the whole heart was by Harrison et al. [33], who attached multiple electrodes to a custom contour-fitting material, a ‘heart sock’, that enabled the acquisition of EP data across the surface of a canine heart on a beat-by-beat basis. These early heart socks contained arrays of up to 52 recording electrodes, and they could be quickly and easily applied to the heart to acquire data simultaneously from multiple points on the epicardium. The resulting activation maps could then be combined with known electrode spacing to assess CV. In subsequent years, more and more electrodes have been fixed to such heart socks to obtain high-density signals, and automated imaging techniques have been used to visualize electrode positions relative to the cardiac surface [34,35]. This technique has even been applied to patients undergoing open-heart surgery [36], using an epicardial sock with 240 electrodes and speckle-tracking transesophageal echocardiography for motion mapping. Measurements using MEA are usually performed epicardially, but endocardial and even dual endo-epicardial mapping with two MEAs is feasible, enabling insight into transmural conduction patterns [37].

Several issues limit the utility of MEA and heart sock recordings. In contrast to OM, contact electrodes record extracellular potentials. This means that, while the timing of activation and (less well) repolarization can be tracked locally, the acquired data contains no information on AP shape. Secondly, contact recordings involve a high degree of invasiveness, requiring open-chest access to place an MEA onto the heart. Thirdly, the beating of the heart can induce displacements between recording electrodes and the underlying myocardium. Ideally, materials that are soft and highly stretchable would be used so that they impart no force on the heart but also ensure close contact of electrodes with the myocardium even while beating. These are typically realized through semiconductor-based designs built on 3D, thin elastic membranes, custom-formed to match the shape of an individual heart [38,39]. Such systems provide conformal interfaces to all points on the heart, with robust contacts enabled by the elasticity of the membrane. This robust contact means that intrinsic CV may be measured as the electrodes should remain approximately stationary relative to the underlying anatomy. Some errors may result from the heterogeneous nature of contraction, which means the relative position between electrodes and myocardium is unlikely to remain completely constant. Furthermore, the requirement for highly individual customization means this system cannot easily be re-used for different hearts, increasing measurement-associated costs and limiting the broad application of this technique to basic and clinical cardiology research.

### 2.3. Intracardiac (Catheter) Electrograms

The most commonly used clinical technique for CV measurement is intracardiac, catheter-based mapping. Catheters equipped with at least two, but in some cases over 50, electrodes collect intracardiac electrograms from different locations in the chamber of interest. Similar to MEA recordings, intracardiac electrograms collect extracellular potentials. Information, such as local signal size and timing, is used to identify critical regions that are potentially responsible for the development and maintenance of arrhythmias, and they have been used in basic, translational, and clinical research to great benefit [40]. 

**Figure 3:**
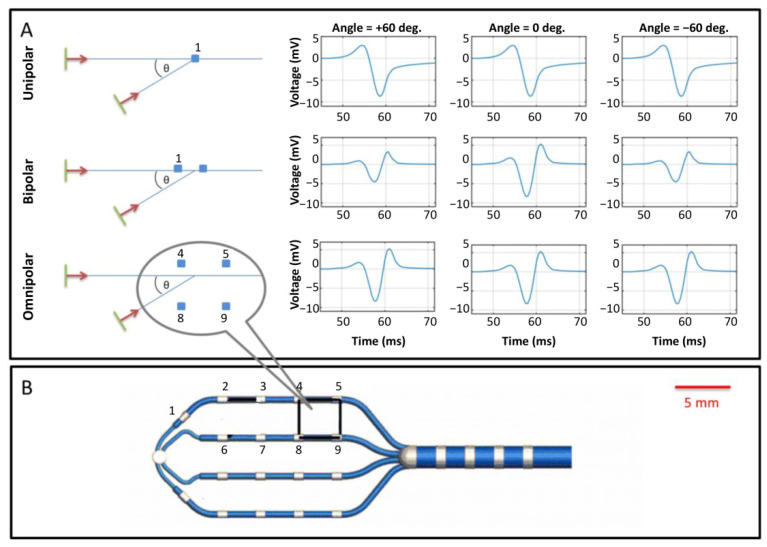
Schematic of intracardiac electrogram recording. (**A**) Illustration of directional ambiguity and amplitude dependence of unipole, bipole, and omnipole. Unipolar signals are independent of a direction of wavefront propagation (top row). Bipolar signals depend strongly on the direction of wavefront direction but cannot distinguish between opposing directions (middle row, compare +60° and −60°). Omnipolar recordings provide identical waveforms regardless of direction (bottom row); however, unlike unipolar recordings, they also provide the direction of propagation (not shown). (**B**) An omnipole design, here consisting of a planar array of 18 electrodes on four splines with a same-spine inter-electrode spacing of 4 mm, and four more electrodes along the catheter’s shaft. Among the many possible electrode pairings, one convenient arrangement consists of a 3 × 3 arrangement of nine rectangular pairings, one of which is illustrated here as 4,5, 8, 9. The planar array inter-electrode spacing of this design is 4 mm. The figure is reproduced with permission from Deno, D.C. et al. Orientation-independent catheter-based characterization of myocardial activation. *IEEE Trans. Biomed. Eng.* **2017**, *64*, 1067–1177, doi:10.1109/TBME.2016.2589158 (accessed on 18 May 2021).

Standard cardiac mapping systems record both unipolar and bipolar electrograms. Unipolar electrograms are signals from a single electrode with a remote reference electrode. They are, therefore, non-directional and sensitive to low-frequency noise, such as far-field or motion-induced recording artifacts [41,42,43]. Conversely, bipolar electrograms record the voltage difference between two electrodes on the catheter and provide more localized measurements of myocardial EP. Although bipolar electrograms are less susceptible to low-frequency noise, they are strongly influenced by the direction of wavefront propagation relative to the spatial positioning of the electrodes (Figure 3A) [41,42]. Bipolar measurements also depend on other parameters, such as electrode spacing, electrode size, and wavefront velocity [44,45,46,47,48]. Most notably, at a single location or orientation, neither bipoles nor unipoles can efficiently provide wavefront characteristics, including velocity and direction. One possible solution is to build an activation map by measuring multiple points; however, this takes time and requires stable EP, as measurements are sequential. Furthermore, when there are multiple local activation pathways (e.g., around scar tissue), recorded electrical signals can be fractionated and present challenges for reliable activation time marking and determining CV (see [49] for a review of the methods and challenges involved with annotating activation time) [49].

In practice, there are temporal uncertainties in the activation timing of up to several milliseconds due to local heterogeneities of activation, noise, and low-frequency baseline drift. Bipolar signals are less sensitive to noise due to the suppression of noise occurring equally at each electrode (so-called common-mode noise), and filtering strongly reduces baseline drift. However, the strong impact of the direction of wavefront propagation relative to the bipolar orientation (Figure 3A) can lead to errors. Although the resulting activation time maps are often reasonable over large regions and time intervals, they can generate locally uncertain and ill-reproducible characterizations of electrical activation, rendering local CV measurements unreliable.

A number of approaches use combinations of bipoles, unipoles, and temporal annotation criteria for EP mapping, typically by utilizing automatic processing algorithms to improve signal quality [50,51]. However, local electrical activation time values must be collected over a long duration and over large areas of the myocardium to acquire a broadly accurate depiction of wave propagation. This low-resolution procedure is time-consuming, as it requires initial spatial mapping and subsequently post-hoc processing and activation analysis of the acquired data, without any options for live imaging.

To address the challenge of accurate local estimates of EP and CV, some studies have used an omnipolar mapping approach [52]. Omnipolar intracardiac electrograms are an evolution of the bipolar configuration, where numerous electrodes are used to generate multiple bipoles in different orientations (Figure 3B). They provide local measurements similar to bipolar recordings but are less affected by the direction of wavefront propagation (Figure 3A). By using a local traveling wave model of propagation, the measurements resulting from omnipolar recordings give the direction of wavefront propagation, as well as signal amplitudes and timings that are independent of this direction [53]. Omnipolar electrograms provide local EP measurements, such as maximum extracellular voltage, CV, and direction, with minimal artifacts, allowing electrophysiologists to generate maps that accurately represent myocardial EP.

Nevertheless, the omnipolar approach has several limitations. A central assumption of this approach is that the myocardial tissue under the catheter is approximately flat. While in some locations, such as parts of the interventricular septum, this assumption is viable, in most locations of the heart, it is not, limiting the accuracy of CV measurements. Furthermore, as with all catheter mapping-based approaches, omnipolar mapping uses a stationary representation of cardiac anatomy, so measurement accuracy is affected by motion.

### 2.4. Electrocardiographic Imaging

Routine non-invasive detection and diagnosis of cardiac electrical activity are currently conducted with a 12-lead ECG. This well-established test is part of routine medical practice. However, the technique records the cardiac electrical activity as a projection on the surface of the body, not from the heart directly. Therefore, it has limited spatial resolution to locate arrhythmic activity in the heart. In recent years, ECGI has emerged as a new approach for the non-invasive acquisition of cardiac EP indicators, including CV. This technique brings together traditional body surface ECG with structural imaging and computational modeling to overcome some of the limitations of the traditional ECG. ECGI can provide high spatial resolution maps of cardiac EP projected onto the surface of the heart. 

**Figure 4:**
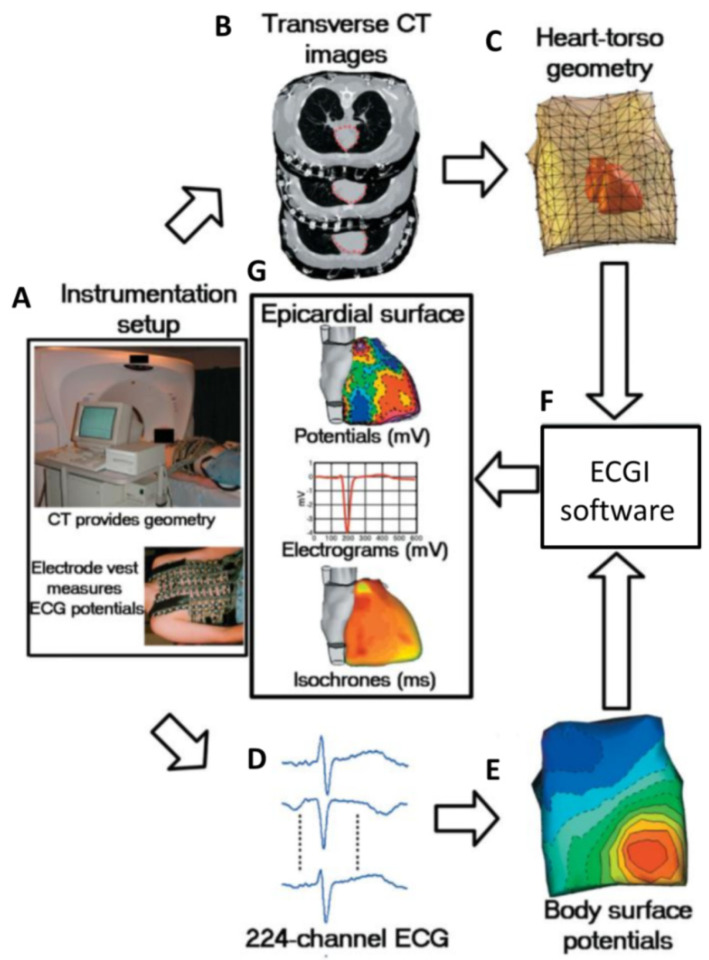
Block diagram of ECGI. (**A**) Photographs of instrumentation setup. (**B**) CT transverse slices showing heart contours (red) and body-surface electrodes (shiny dots). (**C**) Meshed heart-torso geometry. (**D**) Sample ECG signals obtained from the mapping system. (**E**) Spatial representation of body surface potential mapping. (**F**) ECGI software package. (**G**) Examples of non-invasive ECGI images, including epicardial potentials, electrograms, and isochrones. Figure reproduced with permission from Ramanathan, C. et al. Non-invasive electrocardiographic imaging for cardiac electrophysiology and arrhythmia. *Nat. Med.* **2004**, *10*, 422–428, doi:10.1038/nm1011 (accessed on 20 May 2021).

Briefly, a vest that contains 252 electrodes is placed on the torso of a patient and connected to the ECGI data acquisition system. A high-resolution CT scan is then performed to define cardiac anatomy and location relative to the positions of each electrode on the torso. Atrial and/or ventricular geometry are reconstructed to generate a 3D mesh. This model is then used to project unipolar signals, represented by virtual nodes, onto the epicardial surface. The signals collected are post-processed, based on mathematical reconstruction algorithms [54], to produce EP maps, such as for activation, voltage, isopotential, and voltage phase (Figure 4). Thus, ECGI integrates body surface electrical potentials and anatomical information to non-invasively define local cardiac electrical signals (electrograms) over the full surface of the ventricles [54,55]. Using the relative timing of the constructed electrograms, activation sequences (isochrones) can be constructed, and the propagation of activation wavefronts can be assessed, which can then be used for the calculation of CV. In contrast to catheter mapping, ECGI acquires data from the whole heart simultaneously, allowing one to capture dynamic events, as well as static EP. Furthermore, the EP is mapping onto the anatomical surface of the heart that has been obtained across the contraction cycle and in a contact-free manner, ensuring accurate spatial mapping. 

However, the utility of ECGI is limited in several ways. ECGI is also an extracellular measurement, and therefore, does not provide AP information. Cardiac signals are attenuated and combined crossing the thorax, resulting in low-pass filtered signals at the recording electrodes on the torso. Additionally, the reconstruction of the cardiac signal from torso signals can generate large errors in projections to the epicardium as a result of small errors in the signals measured at the torso (measurement noise, electrode position, etc.). Those limitations have been assessed in a number of studies comparing catheter mapping and ECGI [56,57,58]. These studies typically show a poor correlation between the two techniques. ECGI predicted significantly fewer epicardial breakthroughs, and localization discrepancies were often more than 20 mm. While catheter mapping is seen as the current gold standard in clinical practice, it suffers from a number of limitations, as outlined above, and so it is unclear which technique is showing the true state. ECGI holds clear promise to improve measurements of EP and CV; however, remaining uncertainties regarding accuracy must be taken into account when considering its applicability.

### 2.5. Limitations of Current CV Measuring Techniques

Many of the current CV measurement techniques described above suffer from similar limitations. Whether the measurement is made at the endocardium, epicardium, or body surface, they are typically measuring extracellular voltages and not transmembrane signals (with the exception of optical mapping), meaning repolarization is often poorly seen and AP shape not at all. While for CV measurements, this is not problematic, when considering arrhythmogenicity, the APD/repolarization is necessary to assess the vulnerability to re-entry. Most methods also measure extrinsic rather than intrinsic CV (unless material point tracking is used), which limits the fidelity of measurements. In addition, exact conduction paths are rarely known due to the transmural propagation of excitation. For example, if the endocardium is stimulated, the resulting CV at the epicardium may be higher than that recorded when stimulating a point on the epicardium. Similarly, it is hard to be sure whether the measured CV reflects just the underlying myocardium or also occurs via activation of the fast conduction system [59]. 

## 3. Physiological Factors Affecting CV

The heart is a highly complex mechanical pump, passively and actively regulated by mechanical, electrical, and endocrine factors. In general, myocardial conduction is dependent on membrane excitability and passive tissue resistance. Membrane excitability is determined by the availability of voltage-gated sodium channels (I_Na_), whereas resistance is a function of intra, extra, and intercellular resistances. In a healthy heart, key factors affecting CV include the size, shape, and configuration of myocytes and non-myocytes, as well as the connective tissue, cellular volume conductors and gap junctions (GJ). Furthermore, anisotropy and inhomogeneities in the spatial (or in some cases temporal) distribution of these factors are also important. In this section, we focus on physiological factors affecting CV.

### 3.1. Geometric and Intra-/Extracellular Factors

The size and shape of cardiomyocytes vary between species [54], and in the same species, it changes with development [60,61]. In addition, cell size is not constant even across a single heart, which may have significant effects on cardiac EP [60,61,62]. Theoretical and experimental studies have shown that cell geometry can affect CV. In a simplified mathematical model of a cylinder of cardiomyocytes, CV is approximately proportional to the square root of the cylinder diameter [63]. In the multidimensional myocardium, the CV of flat waves decreases along with an increase in the ratio of the cell membrane surface/volume ratio [63].

The elongated shape of the myocytes, as well as intercellular coupling occurring mainly at the cell poles, results in a higher apparent resistance in the transverse direction than in the longitudinal direction. This is called electrical anisotropy. This anisotropy applies both to the extracellular and intracellular spaces of cardiomyocytes. The intracellular resistance is higher in the transverse direction than in the longitudinal direction. Anisotropy in the intracellular space ranges from factors of 5 to 10 [64,65] and mainly results from cardiomyocyte shape. The uneven extracellular distribution of fibrotic material, typically collagen fibers, and non-myocytes, such as fibroblasts, should also be taken into consideration. There is increasing evidence that non-myocytes can alter EP. Originally, fibroblasts were considered to be electrically silent and insulated from cardiomyocytes, but it is now widely accepted that that passive electrical propagation of cardiac excitation via non-myocytes is possible. It has been shown that functional heterogeneous myocyte-fibroblast coupling is sufficient, in vitro, to synchronize spontaneous beating in myocytes over distances of up to 300 μm by fibroblasts only [66]. Functional myocyte-fibroblast coupling (via connexin 45) has been reported in situ for sinoatrial node pacemaker tissue [67]. In addition, myocyte-fibroblast coupling may present in post-infarct scar tissue, and tunneling nanotube connections between myocytes and non-myocytes in scar border tissue is another possible substrate for electrical cell coupling [68,69]. It should be noted that fibroblasts can slow down CV [70] and CV via non-myocytes tends to be much slower, increasing the likelihood of a substrate that can support re-entry. 

Cable theory predicts that decreasing the conductivity of the extracellular space should decrease cardiac conduction velocity [71]. However, changing the extracellular space is often accompanied by changes in cell volume, i.e., edema, or dehydration. Thus, the effects of extracellular space on electrical propagation are complex and are not easily predicted. It remains a challenge to dissect intra versus extracellular conductance and anisotropy in intact cardiac tissue since, due to transmembrane factors, the measured extracellular current between two electrodes will always contain an intracellular component.

**Figure 5:**
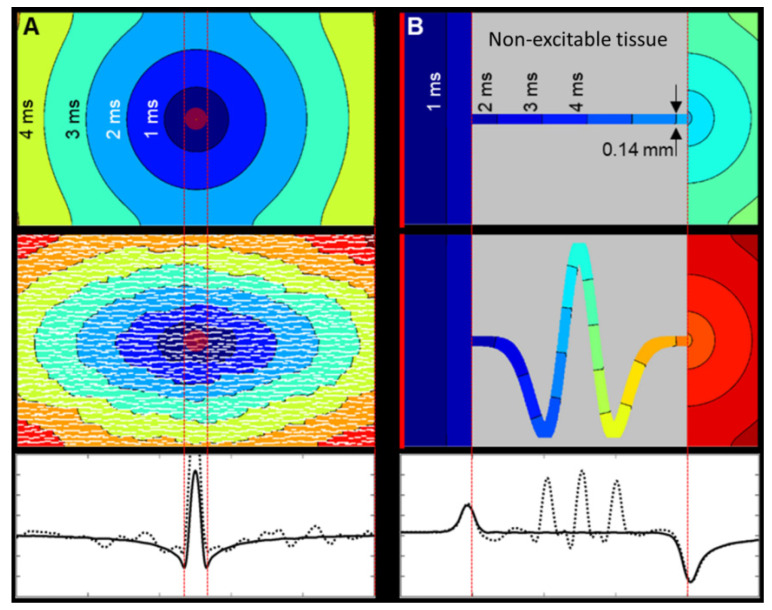
Geometric factors affecting source-sink loading and its effect on CV. Isochrones are separated by 1 ms. (**A**) Focal electrical activation (red central disk), simulated in a simple rectangular 2D tissue model with common membrane dynamics and isotropic electrical conductivity (see Appendix A). (Top) Isotropic tissue. Single vector longitudinal CV is 0.54 m/s and transverse CV is 0.56 m/s. (Middle) Tissue with explicit non-conducting cleft space between layers of cells causes naturally anisotropic activation spread, with a longitudinal single vector CV of 0.57 m/s and reduced transverse CV of 0.28 m/s (i.e., ratio of about 2:1 [76]). (Bottom) Multi-vector CV along the central axis shows the similarity between the isotropic (solid) and discontinuous (dotted) domain CVs. CV is the lowest at the edges of the central stimulus site, as the curvature of the activation wavefront is greatest. (**B**) Planar electrical activation (starting at the left-hand edge: red line) in a simple tissue model of constriction and expansion with Luo–Rudy membrane dynamics and isotropic electrical conductivity. (Top) Simple tissue tract, crossing non-excitable tissue. CV increases upon entering and decreases upon exiting the constriction as a result of step-changes in the source-sink ratio. Single vector (left to right) CV across the whole model is 0.5 m/s, and within the constriction itself, it is 0.61 m/s. (Middle) Tortuous tissue tracts add additional distance to the paths, and local CV is perturbed from a straight path scenario. The apparent single vector CV across the whole model is 0.26 m/s, and across the tract itself, it is 0.65 m/s. (Bottom) While the multi-vector CV before constriction and after constriction in the tortuous model (dotted line) is similar to the straight tract (solid line), local CV along the curved path is regionally increased as the tight bends locally to serve as a ‘shortcut’ (non-consecutive activation of all path elements). In spite of this, activation of the downstream wide tissue segment is delayed, because of increased path length, reducing apparent overall CV.

Interestingly, even relatively simple models of electrical behavior in cardiac tissue can recreate typical features of CV arising from underlying tissue structures. For example, Figure 5 shows two scenarios of (A) focal activation in a rectangular tissue layer and (B) planar activation moving through a narrow tract of myocytes connecting larger tissue regions. The simple reaction-diffusion models employed have isotropic electrical conductivity and commonly used membrane dynamics [72] (see Appendix A for detail). In continuously connected tissue (Figure 5A), focal activation moves concentrically—initially slower, as activation front curvature (end hence sink-source ratio) is high [73], and then faster as curvature decreases. In tissue with explicit non-conducting cleft spaces (typical of interstitial fibrosis developing with aging) between layers of cells (Figure 1B), transverse activation pathways become elongated, causing CV anisotropy [9]. Structural remodeling of post-myocardial infarction can include surviving tortuous tracts of excitable myocytes through otherwise non-excitable tissue (such as patchy fibrosis or compact scar) [74,75]. As a planar activation wave approaches the constricted tract (Figure 5B), CV increases, but exiting at the expansion point, it decreases dramatically due to the sink-source mismatch causing a large electrical load on a small group of activated cells. The downstream reduction of a CV within the expansion can occur over a considerable distance but eventually returns to normal tissue speed. Furthermore, the putative presence of a tortuous tract within the non-excitable region elongates the path length [75], causing later activation at the downstream expansion and, hence, apparent slowing of CV (although CV within the tract itself may actually increase overall). Tissue remodeling involving interstitial fibrosis can alter activation anisotropy and CV, and non-excitable tissue with surviving tortuous activation pathways can affect CV over considerable distances from sites of breakthrough. These tissue structure scenarios showing altered CV, exposed by simple models of geometric features and encoding source-sink biophysics, are often characteristic of arrhythmic substrates [9,75].

### 3.2. Gap Junctions and Connexins

In order to elicit an AP in a cell, depolarizing current from neighboring activated cells must be delivered. The activated cell functions as a current source, while the yet-to-be-activated cell forms a current sink, with the voltage difference between cells driving the inter-cellular current. When there is a mismatch between the current provided by the source compared to that needed to rapidly trigger an AP in the sink (so-called source-sink mismatch), slowing or even blocking of conduction occurs [12]. The current transfer is accomplished mainly through GJ channels, potentially with a contribution via the extracellular space in closely opposed membranes with high densities of sodium channels (ephaptic coupling) [77]. Since the discovery of cardiac GJ, it has been established that they provide a low-resistance pathway for electrical propagation [78]. A GJ channel consists of two hemichannels (connexons) formed in each cell. Each hemichannel is composed of six protein subunits called connexins. In working myocardium, the predominant connexin isoform is Cx43, which is found in ventricles and atria. 

Cardiac conduction occurs primarily via cardiomyocytes. The CV of cardiomyocyte-driven conduction is determined by the speed of conduction within cells and between cells. GJ resistance is higher than cytoplasmic resistance; thus, impulse propagation through the cell is faster than across the intercellular GJ [79]. Therefore, GJ is expected to be the main determinant of CV in intact myocardium. Studies performed with Cx43 gene knockout mice have shown CV slowing only when Cx43 deletion was virtually complete [80,81]. This finding led to the widely accepted general concept that there is a vast redundancy of myocardial GJ coupling. However, more recently, an increasing number of studies suggest that CV depends quite closely on GJ levels [82,83]. One study which measured CV and GJ resistivity found them to be directly proportional when using oil-gap impedance and microelectrode techniques before and during pharmacological GJ uncoupling with 20 µmol/L carbenoxolone [84]. 

Interestingly when examining the safety of conduction, i.e., whether or not failure of conduction will occur, in a mathematical model, it was found that the reduction of CV may increase or decrease safety depending on the mechanism by which slowed conduction occurred. When GJ coupling was reduced, this lowered CV but increased safety until very low conduction velocities, whereas if membrane excitability was reduced instead, both CV and safety were lowered [85]. This effect is likely due to altered source-sink relations with altered GJ where excited cells may couple to fewer cells and so decrease the sink, thereby ensuring excitation despite the reduced coupling strength.

Although the question of whether a continuous relationship between GJ and CV exists varies from study to study, it is undisputed that CV is strongly dependent on GJ. That is, the regulation of GJ conductance can alter CV. The status of GJ channels, such as open or closed, or even the complex conductance state of a single GJ [86], as well as the number of GJ involved in conduction, all contribute to CV variations.

### 3.3. Mechanical Loading

Mechanical changes can evoke EP alterations in cardiomyocytes through the process of mechanical-electrical feedback. Studies performed in isolated rabbit hearts have shown significant and reproducible CV slowing at moderately increased diastolic filling pressures (up to 30 mmHg) compared to unloaded preparations [87]. CV slowing was also seen in isolated mouse hearts, as well as cultured myocytes [88]. Ventricular loading may activate stretch-activated channels, in particular non-selective stretch-activated cation channels, which partially depolarize the membrane at rest [89]. If depolarization is sub-threshold, this could increase excitability (difference between membrane potential and threshold for AP initiation) and hence CV. More pronounced or sustained depolarization would inactivate fast sodium channels, which reduces upstroke velocity and CV. The combination of these interactions may result in a biphasic response to stretch. However, mechanically-induced CV slowing is seen even in the presence of fully blocked non-selective stretch-activated cation channels [90]. Mills et al. further showed that moderate tonic changes in resting membrane potential have little effect on CV, as CV was not significantly altered upon variation of perfusate potassium concentration in volume-loaded Langendorff-perfused hearts [90]. Electron microscopy studies had previously shown that stretching of the ventricular wall leads to the extension of sarcolemmal folds and incorporation of submembrane cavities into the myocardial cell membrane [91], thus resulting in a significant increase in cardiomyocyte membrane capacitance [92]. Mathematical modeling found that cardiomyocyte stretch caused an increase in effective space constant and effective membrane capacitance, which led to a decrease in CV [90]. Mechanical loading thus acts as a key regulator of CV, although whether this occurs on a beat-by-beat basis or only on longer time scales remains unclear. 

## 4. Pathological Factors Affecting CV

EP alterations, also referred to as electrical remodeling, occur in response to pathological changes. In this section, we focus on the effects of specific pathological factors on cardiac CV.

### 4.1. Aging-Associated Changes 

Aging is not a disease. It is associated, however, with tissue remodeling, including reduced cardiomyocyte functionality and increased connective tissue content. This remodeling increases the incidence of cardiac arrhythmias and the risk for sudden cardiac death [9]. Slowing CV with age has been described in rabbits [93], mice [94], dogs [95], and humans [96,97] and has been attributed to increased fibrosis and reduced intercellular coupling in the aging myocardium. Aging-related ventricular CV slowing and increased conduction anisotropy (Figure 5A), has also been associated with alterations in locally prevailing cell orientation and myocardial sheet structure [93]. Furthermore, these structural alterations may also provide a substrate for re-entry and thus increase susceptibility to arrhythmias. Aging-related changes to the myocardium also include alterations in transmembrane ionic currents and thus CV. APD is prolonged in the aged myocardium. This is due to increased late Na^+^ current (*I*_NaL_) and a reduction of voltage-gated outward K^+^ (Kv) currents [98]. While human APD is difficult to measure directly, there is evidence of age-dependent increases in the surrogate corrected QT interval [99,100].

### 4.2. Heart Failure

CV slowing is often seen in heart failure (HF). A study recorded high-resolution optical AP from epicardial and endocardial surfaces of perfused wedge preparations, isolated from the left and right ventricles of normal and failing dog hearts. It is reported that HF was associated with a significant prolongation (by 33%) of the QRS duration (the time for ventricular depolarization) and significant (>20%) slowing of subepi and subendocardial conduction in both ventricles. [101]. This suggested that CV slowing in HF was not related to reduced excitability, as AP upstroke velocity was not altered by HF. Consequently, CV slowing in HF may be attributed to altered cardiomyocyte geometry (i.e., aspect ratio), as well as alterations in GJ. The latter was verified by histochemical experiments: Cx43 expression decreased by more than 40% in the subepi and subendocardial myocardium of the left ventricle [101]. In addition to the downregulation of Cx43 expression, its distribution is also disturbed [102]. Increased lateralization of Cx43 was observed. This is expected to reduce the anisotropy of impulse propagation in the heart, but not all redistributed Cx43 may be functional, as no changes in anisotropy—which might have been beneficial for improving EP—were observed [101]. Another factor causing CV slowing may be alterations to the laminar structure and/or subendocardial emergence of patchy fibrosis, affecting activation pathways. For example, in rats with spontaneous hypertension on the verge of HF, increases in arrhythmia susceptibility were due to increases predominantly in subendocardial patchy fibrosis [103].

### 4.3. Hypertrophy

Cardiac hypertrophy is accompanied by electrophysiological remodeling and an increased risk of severe arrhythmias [104]. This is partly attributable to alterations in cellular EP, such as remodeling of potassium channels. Increased levels of Cx43 and increased Cx43 lateralization have been found in cardiac hypertrophy [102,105]. It is difficult to assess experimentally whether cellular hypertrophy alone has pro-arrhythmic effects. However, cellular hypertrophy results in an enlargement of cardiomyocyte length and/or diameter, both of which influence CV and CV anisotropy [61,106]. Additionally, mathematical models have shown that increased cell size increases the capacitive load, contributing to discontinuities of AP propagation. This makes the relationship between GJ conductance and CV steeper, increasing the risk of conduction block due to uncoupling and/or source-sink mismatch [106]. This suggests that, in addition to the associated cardiac disease, cardiomyocyte hypertrophy itself may form a pro-arrhythmic substrate.

### 4.4. Ischemia

Ischemic heart disease is associated with pronounced, regionally heterogeneous changes in CV [107] due to changes in active and passive membrane properties [108]. Generally, the electrical impedance of myocardial tissue increases after coronary artery closure [109], leading to CV slowing. Immediately following interruption of coronary flow, there is an initial increase of approximately 10–25% in tissue impedance and thus CV, due to collapse of the microvascular system [109]. The second increase in impedance and CV is seen after about 15 min, and attributed to the closing of GJ, i.e., a decrease in intercellular conductivity [109]. 

Prolonged ischemia induces a rise in extracellular K^+^ concentration ([K^+^]_o_), which acts as both a trigger and a substrate for CV alterations and arrhythmias [109]. Increased [K^+^]_o_ alters the resting membrane potential of cardiomyocytes, reducing the availability of I_Na_ [110,111,112], and the reduced excitability also leads to reduced CV.

Although many factors change simultaneously during the early stages of ischemia, several individual factors that uncouple GJ have been identified. Ischemia itself and ischemia-induced catecholamines lead to an increase in free cytoplasmic calcium concentration [Ca^2+^]_i_, which is associated with the uncoupling of GJ and CV slowing or even conduction block [113]. This process is enhanced by the acidosis that occurs during ischemia, which sensitizes GJ to [Ca^2+^]_i_ [114]. As ischemia progresses, acidic metabolites (e.g., lysophosphoglycerides, lactic acid and arachidonic acid) accumulate preferentially in the intercalated disks of ischaemic cells, which also decreases the conductance of GJ [115]. 

A scar may form subsequent to an ischemic event, depending on the severity and duration of ischemia. Arrhythmias preferentially arise from the border zone of the scar, which may result from differences in electrotonic load at the scar edges as a result of regions within the infarct border zone where tracts of preserved myocytes have much reduced cell-to-cell coupling (see Figure 5B) [72]. A 3D geometric modeling study simulating active propagation showed large spatial gradients in electrotronic load in the border zone, corresponding to structural remodeling. These gradients in electrical load lead to variations in the effective refractory period of the border tissue. [116]. Decreases in APD, CV, and upstroke velocity were observed in regions of increasing electrotonic load. This study also demonstrated that focal stimulation in regions with large effective refractory period gradients can cause unidirectional conduction block, providing a potential mechanism for arrhythmogenesis [116].

CV changes in ischemia occur both from the initial ischemic event but also the subsequent electrical and structural remodeling that occurs. These effects tend to compound, greatly enhancing arrhythmogenesis. 

## 5. Compounds Affecting CV

Cardiac CV can be influenced by various compounds. In this section, we will consider compounds for experimental studies on CV, as well as clinical drugs that alter CV.

### 5.1. Experimental Compounds

The effect of blebbistatin on CV is of interest due to its use for excitation-contraction uncoupling. In a study of in vivo Langendorff-perfused pig hearts, significant slowing of CV and prolongation of APD were found after 30 min of blebbistatin application [29]. Similar results were also observed in an isolated rabbit heart model [28]. The latter study also noted that the relative prolongation of the APD was more significant at higher frequencies, and that the cycle length of VF was lower in the normal heart compared to after the application of blebbistatin (0.7 to 2.8 µM) [28]. However, the opposite result was observed in co-cultured neonatal rat ventricular cardiomyocytes and myofibroblasts, where blebbistatin (5 to 10 µM) was observed to dramatically increase longitudinal and transverse CV [117]. These conflicting effects may result from changes in metabolic state, as mentioned in Section 2.1, which are more important in a whole heart than in cell culture.

Alteration of CV can be achieved through the use of GJ uncouplers. Carbenoxolone (CBX) produces inhibition of GJ without a clear selectivity for particular connexin subtypes [118]. CBX has been found to uncouple GJ by over 95% at 2 mM, and the uncoupling effect is of rapid onset and is readily reversible [119]. Heptanol belongs to the long carbon chain n-alkanols family and also uncouples GJ [120]. In Langendorff-perfused mouse hearts, heptanol was found to reduce CV and thus increase ventricular arrhythmogenicity even at low concentrations (50 μM) [121]. The uncoupling effect of heptanol was also found to be reversible when used in cultured rat ventricular cardiomyocytes [122]. Peptide 5 is a connexin mimetic peptide that can reversibly uncouple GJ in a concentration- and time-dependent manner [123] by acting on extracellular loop two of Cx43, adjacent to its matching sequence within the protein [124]. At low concentrations (~10 μM), peptide 5 blocks Cx hemichannels; however, at higher concentrations (~100 µM), it will also uncouple GJ [124]. Palmitoleic acid is another commonly used GJ uncoupling agent and its GJ uncoupling effect is also in a dose and time-dependent manner [125], with uncoupling occurring at 5 µM in rabbit hearts [126]. By uncoupling GJ in rabbit papillary muscles using gradient concentrations of palmitoleic acid, it was found that palmitoleic acid dose-dependently altered the epicardial activation pattern, increased the dispersion of the epicardial activation recovery intervals, and prolonged the atrioventricular conduction time [126]. The GJ blockers all work in a dose-dependent and reversible manner; however, with very different dosing. These compounds can thus be used to better understand the role of CV in arrhythmogenesis.

### 5.2. Clinical Compounds

CV is reduced with decreased cardiomyocyte excitability. Therefore, some sodium channel blockers also cause a reduction in CV. Lidocaine and quinidine are commonly used drugs in clinical practice to treat arrhythmias. As Class I antiarrhythmics, they share the characteristic of affecting the upstroke of AP, but their effects on APD are different. Lidocaine shortens APD but quinidine prolongs it. Therefore, the effect on wavelength differs, with lidocaine having a greater reduction due to reducing both CV and APD. An in vivo canine study using continuous intravenous lidocaine (0.08 to 0.16 mg/kg/min) infusion found that lidocaine caused rate-dependent decreases in CV that were greater in the longitudinal than the transverse direction. Interestingly, at the shortest pacing cycle lengths and when the pacing cycle length was decreased abruptly, a new steady state of CV occurred by the second beat, rather than gradually occurring over time [127]. This rapid change, which has a similar time course to the sodium-current inactivation, suggests that lidocaine may cause changes in CV via sodium channel inactivation rather than via changes in GJ coupling [128]. Quinidine seems to have a similar effect to lidocaine, with a study in mice showing quinidine significantly reduced CV [129]. Quinidine can decrease AP amplitude, prolong APD to 90% repolarization [130], and also has a reduction in longitudinal CV that is more significant than the reduction in transversal CV [131].

Beta-blockers, such as metoprolol and propranolol, are also commonly used antiarrhythmic drugs. It is well known that they can reduce the sinus rate via blocking beta-adrenergic receptors. In ventricular tissue, adrenaline stimulation can lead to an increase in I_Na_ current through the direct effect of cAMP on sodium channel proteins [132], and the number of functional sodium channels mediated by a cAMP-independent G protein can also increase [133]. Thus, beta-blockers do not directly but indirectly reduce CV by blocking the positive effect of epinephrine on I_Na_.

Halothane is a general inhalation anesthetic used for the induction and maintenance of general anesthesia. It reduces the automaticity of the sinoatrial node and the CV of the AV node by blocking L-type calcium channel [134]. Halothane also inhibits I_Na_ in a voltage-dependent manner [135], decreasing the maximum upstroke velocity of AP and reducing CV [134]. It is worth noting that the halothane concentrations in these studies matched clinical anesthesia. In in vivo studies, the effect of halothane on QRS has different results. Dog and human studies have claimed that halothane administration (0.81 ± 0.06 mM) did not significantly alter the PR interval or QRS width but increased the QT intervals [130,136]. Another study reported that 1.0–2.0% haothane prolonged the QRS width without significantly altering the heart rate, PR interval, or QT interval [137]. In this latter result, the hypotension-induced, reflex-mediated increase of sympathetic tone may have counterbalanced the repolarization slowing effects of halothane.

In addition, there are also chemicals in the human environment that can affect CV. Bisphenol A is used to manufacture polycarbonate plastics and epoxy resins that are used widely in products, such as food and beverage containers, toys, and medical devices. Exposure to bisphenol A concentrations as low as 10 μM can result in atrioventricular delay, while 100 μM can lead to sustained and complete atrio-ventricular block. Bisphenol A concentration-dependently reduces ventricular CV (1–100 μM) and prolongs APD (0.1–100 μM) [138]. Although bisphenol-A concentration in human serum varies depending on environmental exposure, concentrations ranging from 3.46 to 31.19 μM in healthy populations have been reported [139].

Given that the CV can be modified by many compounds, it is important to choose suitable candidates in experimental design and to be aware of possible off-target effects from other treatments, anesthesia, or possible environmental exposure.

## 6. Conclusions and Future Directions

Slowed or heterogeneously altered CV can provide a key substrate for arrhythmogenesis, with slowing increasing the probability of re-entrant arrhythmias by reducing the length scale over which re-entry can occur, while heterogenous CV also increases the heterogeneity of repolarization. Therefore, it is important to explore factors that can influence CV in physiological and pathological conditions and to find techniques for CV characterization that are accurate, reproducible, and suitable for different scenarios to explore cardiac electrical remodeling and arrhythmogenesis.

Improved CV measurement methods are necessary, as is the standardization of how CV is reported. The current measurements outlined in this paper largely measure extrinsic CV based on results derived from changes in extracellular potential at the surface of the cardiac tissue (sub-endocardium or sub-epicardium). Methods to measure intrinsic CV, particularly in the clinical setting, and ideally doing so in 3D on a beat-by-beat basis, are necessary to better understand the link between CV and arrhythmogenesis and identify the risk of arrhythmias. Magnetocardiography, mechanical mapping, and optoacoustic imaging may all serve to do so; however, none are yet in routine clinical use. 

Magnetocardiography enables the measurement of electrical vectors in the heart in 3D, and with recent advances in clinical measurements of atrial and ventricular activation and repolarization without geomagnetic field shielding, it has become significantly more accessible [140,141]. However, (i) it requires additional imaging to project electrical source information to the underlying cardiac structure, (ii) it cannot be used to measure transmembrane voltages, and (iii) it works best in combination with ECG due to different sensitivities to in-plane versus out-of-plane wave propagation [142,143]. 

Mechanical mapping involves the use of imaging techniques, such as ultrasound or MRI, to track the mechanical activation across the heart. Assuming a constant delay between electrical and mechanical activation, it can thus be used as a surrogate read-out for electrical activation and so for CV [144]. However, while CV can be approximated, repolarization cannot be measured, limiting the utility of this for assessing arrhythmia risk in isolation. 

Optoacoustic imaging uses ultrasound to record sound waves resulting from optical excitation. This technique thus has much greater penetration depths than standard optical imaging techniques but retains many advantages of optical imaging. Voltage-sensitive optoacoustic dyes have been described [145]; however, their application to cardiac imaging has not yet been reported, nor has clinical optoacoustic imaging of the heart been performed. 

With the advent of 3D measurements of CV, the role of anisotropy should be further considered. CV anisotropy is necessary in the healthy myocardium, but if altered, it may also be a source of arrhythmogenesis. This is often ignored, particularly when looking at the length scale for re-entry or wavelength: this is a key concept, where usually just the longitudinal conduction is considered. The risk for re-entry is thus simplified to a 1D number, which, while easy to interpret, ignores the 3D nature of the myocardium. An alternative is to use the wave-volume (which is defined as the product of cardiac wavelengths in longitudinal, transverse, and transmural directions). Therefore, 3D re-entrant arrhythmias can be initiated and sustained when the cardiac tissue volume is greater than wave-volume [59]. The use of wave-volume in combination with techniques to measure CV in 3D will allow the development of a more nuanced identification of the risk of arrhythmias and arrhythmia mechanisms.

## Figures and Tables

**Figure A1:**
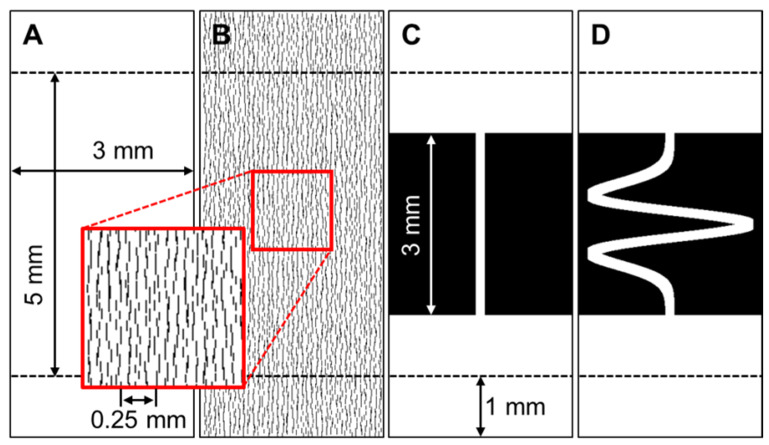
Model domains. Areas in white are viable myocardium, and areas in black are the equivalent of a non-conductive scar. All models are 7 by 3 mm, but for comparison purposes, only the central 5 mm section is used to mitigate top and bottom boundary effects. (**A**) Isotropic domain. (**B**) Laminar cleft domain. Clefts are around 50 μm apart. (**C**) A simple model of a tissue tract crossing the scar region with constriction and expansion. (**D**) A tortuous tissue tract crossing the scar region. The tortuous tract is 9.05 mm in length.

**Figure A2:**
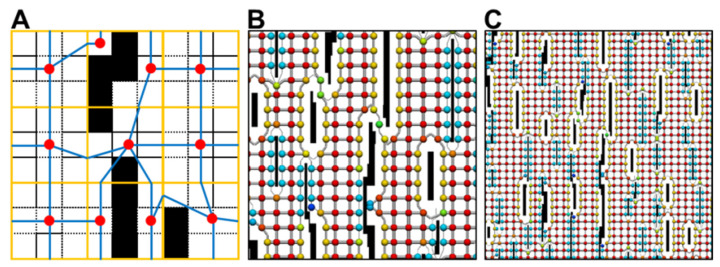
Discretizations of laminar cleft domain. Domain defined by 300 × 700 pixel binary image. (**A**) Domain is split into blocks of 3 × 3 pixels (orange). Each block is abstracted to a center-of-mass node (red) and edge links (blue). These are linked to form a network. Each pixel size is 0.01 × 0.01 mm. (**B**) The discrete network on a larger scale showing how the network forms around the laminar breaks. Red spheres show the maximum area (0.03^2^ mm^2^) associated with a node, and blue spheres show the smallest area. (**C**) A network view from further back.

**Figure A3:**
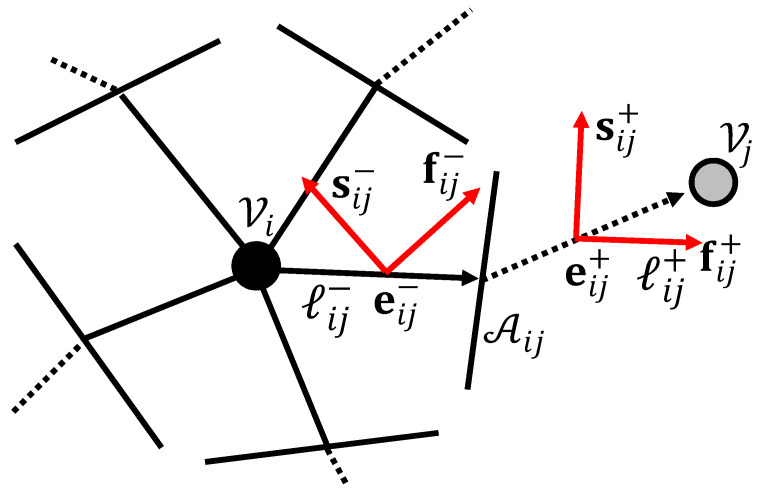
Components of a generic network node–edge–node connection.

## Data Availability

Not applicable.

## Appendix A

### Appendix A.1. Introduction

This appendix describes methods used to construct and solve cardiac activation models for illustrating the effects of laminar discontinuities and cell strands on conduction velocity distributions. Model results are shown in Figure 5.

### Appendix A.2. Model Domains

Four different two-dimensional models were constructed. Model domains are described by the binary images shown in Figure A1. White regions are electrically-viable myocardium, and black regions are inert, for example, scar tissue or cleft spaces. Model 1 (Figure A1A) is an isotropic control domain, and Model 2 (Figure A1B) occupies the same area, but the domain is disrupted by randomly distributed electrically inert breaks aligned vertically (see Figure A1B insert). These breaks model laminar clefts that are prevalent in cardiac tissue and are typically separated by several cell widths [10]. Model 3 (Figure A1C) represents a cell tract (approximately 7–10 cell widths) bridging two larger areas, so there is a constriction entering the tract and an expansion exiting. Model 4 (Figure A1D) has an identical entry and exit to the tract but the cell tract is tortuous and elongated.

The construction of a discrete computational network of nodes and edges is illustrated for Model 2 (Figure A1B) as it is the most complex substrate. The domain images are subdivided into 3 × 3 pixel blocks (Figure A2A), and each block is abstracted to a center-of-mass, and the edge links to adjacent blocks across non-inert edges. The network flows around the inert laminar clefts (Figure A2B), but the network mesh between the clefts remains regular (Figure A2B). For models 1, 3, and 4, the network mesh is mostly regular.

### Appendix A.3. Modeling Electrical Activity in the Networks

The sequence of electrical activation through the networks was modeled using a finite volume discretization of the reaction-diffusion monodomain [146]:

(A1) $$A_{m}C_{m}\frac{\partial V_{m}}{\partial t}-\nabla \left(\sigma \cdot \nabla \;V_{m}\right)=-A_{m}\left(I_{m}-i_{s}\right).$$

This equation is subject to external boundary conditions (with boundary normal b):

(A2) $$\nabla \;V_{m}\cdot \left(\sigma b\right)=0.$$

Here $V_{m}$ is the membrane potential, $A_{m}$ the nominal cell surface area to volume ratio, $C_{m}$ a specific membrane capacitance, σ an effective bulk electrical conductivity tensor, $I_{m}$ the membrane current per unit area (this is conventionally positive for current flowing across the membrane from within the cell), and $i_{s}$ is a stimulating transmembrane current. In the models described in Appendix A.2, isotropic conductivities were specified, so the conductivity tensor became a single scalar value. The discrete network captured any laminar discontinuities.

Equation (A1) can be expressed in a spatially discrete (temporally continuous) form as:

(A3) $$M\frac{\partial V_{m}\left(t\right)}{\partial t}-K\;V_{m}\left(t\right)=-\frac{1}{C_{m}}M\left(I_{m}\left(V_{m},s,t\right)-i_{s}\left(t\right)\right).$$

The matrix M is the mass matrix and the matrix K is the stiffness matrix. The vector of states for the membrane model is given by the vector s. Following integration over a discrete time step from t^n^ to t^n+1^, a first-order split time step approximation to Equation (A3) is:

(A4) $$\left(M-\Delta tK\right)V_{m}^{n+1}=MV_{m}^{n}+\Delta t\;M{\hat{V}}_{m}^{n+1}.$$

The predicted membrane potential arises from the solution of a system of ODEs describing cell membrane dynamics:

(A5) $$\frac{{\hat{dV}}_{m}}{dt}=-\frac{I_{m}\left(V_{m},s,t\right)-i_{s}\left(t\right)}{C_{m}}.$$

The entries in the mass and stiffness matrices are:

(A6) $$M_{ii}=A_{m}C_{m}V_{i}\;M_{ij}=0\;,\forall \;i\neq j\;K_{ii}=-\underset{j}{\sum }\frac{A_{ij}}{\ell_{ij}^{-}+\ell_{ij}^{+}}\frac{1}{2}\left(\sigma_{ij}^{-}+\sigma_{ij}^{+}\right)\;K_{ij}=\frac{A_{ij}}{\ell_{ij}^{-}+\ell_{ij}^{+}}\frac{1}{2}\left(\sigma_{ij}^{-}+\sigma_{ij}^{+}\right)\;,\forall \;i\neq j,$$

with the geometric components as shown in Figure A3. Since the conductivity in the models of Appendix A.2 is homogeneous and isotropic, the $\frac{1}{2}\left(\sigma_{ij}^{-}+\sigma_{ij}^{+}\right)$ term is effectively a constant value.

The network discrete monodomain equations described by Equations (A4)–(A6) were implemented in custom-written code, which was parallelized and tuned for both memory and computational efficiency.

To advance the discrete equations in time, first-order operator-splitting was used, where one solution of the predictor membrane potential Equation (A5) was found for each time step. This is an identical approach to what has previously been used in a finite element discretization [147]. A carefully optimized conjugate gradient method was written to solve Equation (A4). The predictor membrane potential was found by solving an LRd model [148] of membrane dynamics. Using a similar approach to Rush and Larsen [149], some equations were approximated by their closed steady-state form. Other equations were modified as necessary using l’Hôpital’s rule to ensure they remained bound to their limiting values as both numerator and denominator approached zero.

Potentials and activation times were found throughout the network for various stimulation protocols. The parameters used were as follows: time step 0.005 ms, $A_{m}$ 200 mm^−1^, $C_{m}$ 0.01 μF/mm^2^, and σ 0.1 mS/mm. This electrical conductivity is consistent with Hooks et al. [150] and unpublished observations from the same authors.
